# Dietary Habits among Adolescent Girls and Their Association with Parental Educational Levels

**DOI:** 10.5539/gjhs.v5n5p202

**Published:** 2013-07-23

**Authors:** Manijeh Alavi1, Monir Baradaran Eftekhari, Rosemary Noot, Jafar Rafinejad, Ahdieh Chinekesh

**Affiliations:** 1 Center for Development and Cooperation of Research and Technology, Undersecretary for Research and Technology, Ministry of Health and Medical Education, Tehran, Iran; 2 Social Determinant of Health Research Center, University of Social Welfare and Rehabilitation Science, Tehran, Iran; 3 Department of Medical Entomology and Vector Control, School of Public Health, Tehran University of Medical Sciences, Tehran, Iran

## Abstract

**Background:**

Adolescence is a period of rapid psychological development and the appearance of secondary sex characteristics. Changes in facial structure are the most visible manifestation during this period. It is during adolescence period that the importance of optimal nutrients is greatest. Improving the nutrition of teenage girls especially is important girls because consequently will affect the health in future. In present study hypothesis is that improving the nutrition of teenage girls is correlated with the level of parental education and the adolescents’ eating habits.

**Methods:**

In this study, 386 random selected adolescent girls were selected by cluster sampling. We used questionnaire to study the level of knowledge to major nutritional problems and consuming optimal nutrients. Finally, collected data were analyzed by using descriptive techniques and statistical analysis.

**Results:**

According to the results of present study the mean of age, weight and height of the participants were 13/2 years, 159/1 cm, and 52.05 kg respectively. The 48.4 percent of the participants not eat breakfast. The 67.4 percentages of girls daily were consuming bread and cereals, 57.5 fruits and vegetables, 62.7 dairy products, and 27.7 meat and eggs. In addition, 36.3 percentages of these girls consumed sweets everyday as part of their diet. The nutritional knowledge of participating in diet was on the average and common source of information were counselors and teachers at school (36 percentages). The results of our study revealed that there was a significant relationship between variations in level of parental educational level and dietetic safety of offspring (p<0.05). Our conclusion is the interactive education and parental literacy especially is important regarding to the adolescent nutrition and health.

## 1. Introduction

Throughout a person's life, certain events will occur which is of particular importance and is considered as a turning point in their lives (Afaghi et al., 2012). Adolescence is a period of rapid growth and the appearance of secondary sex characteristics (West and McNamara, 1999). Due to rapid physical growth of adolescents, physiologiacl activities are increased and they need more energy to meet increasing demands in comparison to previous developmental period. Breakfast is the most important meal in the dietary plan of an adolescent. Adequate intake of animal and plant sources of protein is vital for adolescence. Vitamins and minerals such as calcium, iron, and iodine must be included in adolescents’ diet. Best sources of vitamins are fruits and vegetables while milk and dairy products are the best sources of calcium (Hallstrom et al., 2012).

Unfortunately, in some countries too little attention has been given to adolescent nutrition. The result of these insufficient attentions is either insufficient or excessive diet. For example, Fidler (2012) showed the in appropriation of adolescents’ dietary habits in Slovenia were associated with the growth problems. In China, the average quantity of protein consumption in children and adolescents in 1991 to 2009 has decreased and caused the deduction of weight and height among adolescents (Lopes et al., 2012). Overby (2012) showed that Norwegian adolescents that consuming appropriate diet and do not omit breakfast have lesser behavioral problems. However, studies in some countries such as South Africa showed decreased consumption of breakfast among adolescents (Lopes et al., 2012). Several studies have stressed adolescents need to understand the importance of nutrition in this stage and have emphasized the importance of educational interventions (Sichert-Hellert et al., 2011; Kersting et al., 2008). In addition, families have important role in creating appropriate eating habits and physical activities. Support and encouragement from families and parental modeling would result to healthy nutrition and the performance of physical exercise or the opposite situations would result to less encouraging results (Menon et al., 2013). Meanwhile, adolescent girls’ nutrition is vital because improving female adolescence nutrition behaviors is an investment for improving health among future generations (Locks et al., 2013; Huffman and Schofield, 2013).

The present study was conducted to assess adolescent girls’ knowledge, their nutritional habits, and its association with parental education.

## 2. Material and Methods

In this study, participants were female students in 3^rd^ year guidance public in the city of Tehran. To estimate the sample size, the total numbers of female students were obtained from the Department of Education and Culture. According to the Morgan statistical formula, 381 samples were taken on the dates. To obtain the population sampling, the random cluster sampling technique was used. The city of Tehran was divided into 5 regions including north, east, west, south, and central district. One school in each district of regions is chosen. In next step, students were categorized according to the related districts. Finally, the information of 62 students in the northern district, 59 students in the central region, 87 in the western region, 109 students in the eastern region and 69 in the southern region were collected. The participants were informed about all ethical considerations and permissions were taken from the participants in this study.

The tool used in collecting data includes three questionnaires constructed by the researcher on knowledge; attitude and performance (KAP) in the area of nutrition. After confirming their validity by experts and respective teachers, test-retest method was used to confirm their credibility. Questionnaires were as multiple-choice questions and participants were instructed to choose only one correct answer. In scoring the level of knowledge, attitudes and method of performance, the total score considered in the questionnaire were calculated and were divided into 5 items: extremely low (extremely negative, extremely incorrect), low (negative, incorrect), moderate (approximately positive, approximately correct), high (positive, correct) and extremely high (extremely positive, extremely correct). Scores obtained by each respondent were categorized in the corresponding level. We analyzed the 386 questionnaires by descriptive and inferential statistical techniques including the analysis of variance and Tukey test. In order to compare mean results (mean age at menarche, mean score for knowledge, attitudes, and performance) one-way Anova and post hoc Tukey tests were used. All statistical procedures were done with the use of the SPSS Inc 17.

## 3. Results

According to the results of this study, the mean of age, height and weight of participations adolescent were 13.2 years, 159.1 cm, and 52.05 kg, respectively. Regarding the parents’ educational level, 39.1% have finished high school and 0.3% has attained a doctoral level.

Majority of the participants (48.4) skipped breakfast while only few (8.3 percent) skipped lunch. Almost 67.4 percent of the girls have expressed that they consumed enough bread and cereals daily and only 2.1 percent have expressed that bread and cereals is not a part of daily diet. More than half of individuals (54 percent) who exclude bread and cereals in their dietary intake have expressed that their reason for not taking these food is “their lack of desire.” The 62.7 percentages of the participants have expressed that they consumed at least (1≥serving) daily of dairies while 4.4 percent expressed the dairies is not a part of their diet. The reason of lack of dairies in diet commonly expressed by “they don’t like to east such food” or weight losing program (9 percent). The 27.7 percent of the girls have stated that they consume one serving of meat and eggs daily while majority of them (53.1 percent) have expressed that they only consume 2 or 3 times serving of meat and eggs per week. Some girls gave reason for consuming meat and egg “family dietary habits and they have to abide with it” (62.8 percent). The 57.5 percent of the participants have stated that they consume at least one serving (1≥ serving) of fruits and vegetables daily while 2.1 percent have stated that they never eat fruits and vegetables.

Regarding the participants consumption of sweets and pastries, 36.3 percent have stated that these foods are part of their daily diet while 8.5 percent have expressed that these types of foods is never been a part of their daily diet. Majority of the reason (39 percent) for not consuming such food is that “they are dieting and are trying to avoid such foods.” In this study, 38.1 percent of the participants have stated that they are on a dieting regimen while 59.3 percent were not on diet. Majority (47.9 percent) believed that dieting is not necessary during adolescent period.

Result of this study showed that participants’ level of knowledge regarding adolescent's nutrition is moderate (41.2 percent). The participants’ attitude in most cases was very positive. As we can see in tables 1-2, dietary behaviors of the majority of participants per evaluation were on the medium level (41 percent).

**Table 1:** Frequency distribution of students’ knowledge on nutrition and nutritional behavior

Level of awareness	Absolute Frequency	Percentage
Frequency distribution of students’ knowledge on nutrition		
Extremely low	26	6.8 %
Low	45	11.7%
Moderate	159	41.2%
High	88	22.8%
Extremely high	68	17.6%
Total	386	100%
Frequency distribution of students’ nutritional behavior		
Extremely incorrect	10	2.6%
Incorrect	18	4.7%
Moderate (fairly accurate)	210	54.4%
Accurate	100	25.9%
Extremely accurate	48	12.4%

**Table 2:** Frequency distribution of students’ attitude towards the importance of adolescents’ nutrition

Type of attitude	Absolute Frequency	Percentage
Extremely negative	4	1%
Negative	9	2.3%
Moderate	64	16.6%
Positive	130	33.7%
Extremely positive	179	46.4%
Total	386	100%

According to the results, the most students (54.4 percent) had moderate nutritional behavior (accurate), and the frequency of students with extremely incorrect nutritional behavior were lowest (2.6 percent). Results of the present study showed that there is a significant relationship between parental educational level and participants’ level of awareness and nutritional performance (p-value < 0.05). In addition, the sources of students’ information were including teachers and schools counselors (36 percent), family (29 percent), friends (25 percent) and media (10 percent) respectively.

## 5. Discussion

In this study, consumption of certain food groups such as fruits and vegetables was lower than expected threshold that is consistent with the study conducted by Zhang (2012) in China. The study conducted by Erinosho and associates in America (2012) has reported the consumption of at least (≤ 5 serving) daily of fruits and vegetables (Erinosho et al., 2012). Also in Kuwait consumption of vegetables and variety of green salads has reduced from 2006-2008 (Zaghloul et al., 2012). In present study, the daily consumption of dairies was reported to be 62.7 percent that is lesser in comparison to Australian adolescents (Baird et al., 2012). Results of vegetable and dairy consumption in our study are more consistent with the result of the study conducted in Georgia by Kherkheulidze and associates in 2012 on high school students. In addition, the consumption of bread and cereal in our present study is also consistent with the study conducted in Georgia by Kherkheulidze and associates in 2012. However, consumption of sweets and confectionaries in Iranian girls are lesser in comparison to Georgian girls. In this study, 52 percent of Iranian adolescent girls eat their breakfast while 48 percent of these girls skip or eat very little breakfast while results of the study conducted in Georgia showed that 62 percent of these girls eat their breakfast (Kherkheulidze et al., 2012). Knowledge regarding nutrition among majority of Tehranian adolescent girls was moderate (41.2 percent) while Georgian girls have poor knowledge regarding nutrition. We should be mentioned however, their study were high school girls (Kherkheulidze et al., 2012). Results of another study conducted in South Africa in 2012 showed that knowledge in nutrition and physical education and diet among high school girls are not enough (Letlape et al., 2010).

In a study conducted on students aging 11 to 18 years in the Healthy Heart Project conducted by Esfahan Medical University of Medical Sciences has reported that these girls have moderate knowledge regarding nutrition (Boshtam et al., 2010). In addition, students’ attitudes towards the importance of nutrition in adolescents and parental education were not found to be significant however; there exist a significant relationship between the students’ awareness and nutritional behavior to their parental educational level (p<0.05). This study and the studies conducted regarding the important role of parents in encouraging their children towards adopting healthy nutrition and performance of exercise during adolescence are consistent (Sleddens et al., 2012; Totland et al., 2013). However, the study conducted by the Cardiovascular Research Center, Esfahan University of Medical Sciences has not found any significant relationships between the nutritional score of the subjects of the study and the level of parental education and this finding is inconsistent with the findings in our study (Totland et al., 2013). However, in the study conducted by Lopez and associates in 2012, did not point out directly the relationship between awareness, nutritional practices and parental educational level but they reported the students having parents with lower educational levels have lower BMI, weight and height in comparison to adolescents’ with parents having higher educational level (Lopez et al., 2012).

In present study, the most important source of education on nutrition among the participants were the teachers and the school counselors (36%) which is consistent to the results of the study conducted by Grand MJ in 2012. In addition, in his study American and Taiwanese adolescents have considered their teachers as an important source of information regarding nutrition, physical education, and health (Grant, 2012).

The present study showed that there is a need to educate and improve the nutritional status of adolescents and the presence of their parents is necessary. As has been stressed in many previous studies, program planning, and policymaking have an important role in improving the life style status of the society where adolescents live (Downs et al., 2012).
